# Communication and Common Interest

**DOI:** 10.1371/journal.pcbi.1003282

**Published:** 2013-11-07

**Authors:** Peter Godfrey-Smith, Manolo Martínez

**Affiliations:** Philosophy Program, The Graduate Center CUNY, New York City, New York, United States of America; University of Washington, United States of America

## Abstract

Explaining the maintenance of communicative behavior in the face of incentives to deceive, conceal information, or exaggerate is an important problem in behavioral biology. When the interests of agents diverge, some form of signal cost is often seen as essential to maintaining honesty. Here, novel computational methods are used to investigate the role of common interest between the sender and receiver of messages in maintaining cost-free informative signaling in a signaling game. Two measures of common interest are defined. These quantify the divergence between sender and receiver in their preference orderings over acts the receiver might perform in each state of the world. Sampling from a large space of signaling games finds that informative signaling is possible at equilibrium with zero common interest in both senses. Games of this kind are rare, however, and the proportion of games that include at least one equilibrium in which informative signals are used increases monotonically with common interest. Common interest as a predictor of informative signaling also interacts with the extent to which agents' preferences vary with the state of the world. Our findings provide a quantitative description of the relation between common interest and informative signaling, employing exact measures of common interest, information use, and contingency of payoff under environmental variation that may be applied to a wide range of models and empirical systems.

## Author Summary

How can honest communication evolve, given the many incentives to deceive, conceal information, or exaggerate? In recent work, it has often been supposed that either common interest between the sender and receiver of messages must be present, or special factors (such as a special cost for dishonest production of signals) must be in place. When talk is cheap, what is the minimum degree of common interest that will suffice to maintain communication? We give new quantitative measures of common interest between communicating agents, and then use a computer search of signaling games to work out the relationship between the degree of common interest and the maintenance of signaling that conveys real information. Surprisingly, we find that informative signaling can in some cases be maintained with zero common interest. These cases are rare, and we also find that the degree of common interest is a good predictor of whether informative signaling is a likely outcome of an interaction. The upshot is that two agents with highly incompatible preferences may still find ways to communicate, but the more they see eye-to-eye, the more likely it is that communication will be viable.

## Introduction

Many theorists have seen communication as a fundamentally cooperative phenomenon [1]–[4]. In an evolutionary context, however, cooperation cannot be taken for granted, because of problems of subversion and free-riding [5]. In the case of communication, these problems include both refusal to share information, and deception, or lying for one's own advantage. If lying is common, there is no point in listening to what anyone says. If no one is listening, there is no point in talking.

In recent work the situation is often sketched as follows: it is easy to see how communication can be viable if there is complete concordance of interests between senders and receivers of signs. Then communication can result in useful coordination and division of labor. There is no mystery about signaling within multicellular organisms, for example, including hormonal and cell-to-cell signaling (although conflicts of interest may arise even here: [6]). In between-organism contexts, the problem of conflict of interest rapidly becomes acute. Special mechanisms are needed to explain how honesty is maintained. The main approach taken in recent years has been *costly signaling theory* [7]–[9]. Intrinsic costs of signaling prevent dishonesty, by differential expense to liars or differential benefits to the honest.

“Cheap talk” models, where signaling has no costs, have seen some development [10]–[15] but have been minor players in recent years. Here we use a novel method to examine ways that informative signaling can be sustained without cost in a range of situations of partial and low common interest. We use a version of the Lewis sender-receiver model [1], [16], and employ a method of sampling and analyzing cases drawn from a large space of games with different relationships between sender and receiver payoffs. We then offer generalizations based on analysis of the sample of cases. The analysis uses coarse-grained measures of common interest between sender and receiver, and attends also to a feature that interacts with common interest: the degree to which payoffs for an agent depend on different acts being produced in different states, the *contingency of payoff* for that agent.

We find that using a simple and intuitive measure of common interest based on comparisons of preference orderings over actions, it is possible, though rare, for informative signaling to be maintained at equilibrium with complete divergence of interests. We then construct a more fine-grained measure of common interest, one that is more demanding in its classification of a case as one of zero common interest, and find that informative signaling with zero common interest is possible in this stronger sense as well. Defining an *information-using equilibrium* as one where the receiver makes use of informative signals to guide behavior, the proportion of games that include at least one information-using equilibrium increases monotonically and rather smoothly with both measures of common interest. (See below, in the *Methods* section, for the equilibrium concept we use throughout the paper.) We then look at the equilibria that support the *highest* amount of information use for a given level of common interest, and again find a monotonic, though less smooth, relationship between degree of common interest and maximum information use. A third analysis, looking at the relationship between common interest and contingency of payoff for sender and receiver (defined below), yields more complicated results.

We conclude that informative signaling can be stable in situations of minimal, even zero, common interest. A combination of *mixed strategies* of signal use by both senders and receivers, and the selective *pooling* of states by the sender, makes possible the extreme cases of this phenomenon. Pooling alone can suffice in cases where divergence of interests is not so extreme. As interests converge, stability of informative signaling becomes easier to achieve. Our model complements other recent work on the adaptive importance of mixed strategies and partially informative signaling in evolution.

## Methods

Our modeling framework draws on Lewis [1] and Skyrms [16]. We assume that the world varies exogenously and has three equally probable states (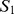, 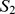, 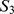). The sender perceives (without error) the state of the world and responds by mapping states to signals (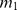, 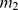, 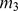). The mapping need not be one-to-one as the sender may “pool” some states, treating them equivalently, and the sender may also probabilistically “mix” signals in response to a given state. The receiver perceives (without error) the signal sent and maps signals to acts (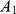, 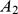, 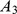), with pooling and mixes possible again. So a combination of sender and receiver rules can be represented as follows:

**Sender:** 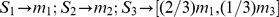


**Receiver:** 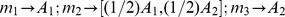


For example, the sender here sends message 1 whenever they see state 1, message 2 whenever they see state 2, and in state 3 they flip a biased coin to send message 1 two thirds of the time and message 3 one third of the time. Both sides receive payoffs as a consequence of the combination of the receiver's action and the state of the world. Sender and receiver payoffs may differ, and can be represented in the form seen in Table 1.

**Table 1:** A payoff matrix.

	*S*_1_	*S*_2_	*S*_3_
*A*_1_	5,0	2,4	0,6
*A*_2_	6,5	0,0	1,5
*A*_3_	0,6	6,6	5,3

The pair of numbers in each cell represent the sender's and the receiver's payoffs, respectively, for a receiver action (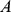) performed in a given state of the world (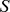).

The payoff matrix defines a preference ordering over acts in each state for both sender and receiver. For example, in Table 1, the preference ordering for the sender in state 1 is [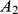
>
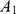
>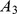], and for the receiver [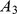
>
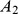
>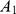]. A simple measure of the degree of common interest in a game tracks how similar the orderings for sender and receiver are, for each state: there is *complete common interest* when sender and receiver have the same preference ordering over acts in every state, and *complete conflict of interest* when these orderings are reversed in every state. Between these extremes are various kinds of *partial* common interest: sender and receiver might agree on the best act in each state, but disagree otherwise; they might always agree on what is worst, but not otherwise; they might agree entirely in some states but disagree in others.

In cases of complete common interest, some consequences for informative signaling are easily seen. With complete common interest, sender and receiver can both receive their maximum payoffs when the sender maps states to signals one-to-one and the receiver uses these signals to guide appropriate actions. This is a *signaling system* in the sense of Lewis [1], and neither party has any incentive to change what they are doing. This state might not be attained by the selection process shaping sender and receiver behaviors, but if it is reached it is stable [17]. With complete conflict of interest, it would appear that signaling cannot be maintained, as any information about the state of the world carried by signals can be used by the receiver to produce acts contrary to the sender's interests, and any sensitivity to signals in the receiver can be exploited by the sender. Exploring the generality of this phenomenon is one aim of this paper. Another is quantifying the relationship between common interest and informative signaling.

The varieties of partial common interest described above do not form a complete ordering. However, a coarse-grained measure of the overall degree of common interest can be constructed by modifying the *Kendall tau distance*. This measure describes the similarity in the ordering of the items in two lists, by counting *discordant pairs* of items across the lists. The first two items in the two lists form a discordant pair with respect to a preference ordering, for example, if in list 1 the first item is preferred to the second item, whereas in list 2 the second item is preferred to the first. We define a measure *C* of the common interest in a payoff matrix of the form in Table 1 by counting the discordant pairs in the sender's and receiver's preference orderings over acts in each state of the world, and then averaging across states and rescaling the results to yield a number between 0 and 1, where 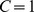 corresponds to complete common interest and 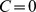 corresponds to complete conflict of interest. In response to results outlined below we also make use of a refinement of 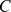; which compares not only the agents' preference orderings of the actions in each state, but also tracks how the agents' payoffs for each action relate to the mean value of the payoffs the agent might receive in that state. (For details see Text S1.) As discussed below, 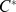 is one among several ways of refining the simpler measure, 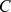, and we do not claim it is best for all purposes.

We also make use of a further description of payoff matrices. For each agent, how much does payoff depend on matching different actions to each state of the world? A simple illustration of the importance of this feature is seen in a case where the receiver has the same best act for every state (has a dominant strategy available). Then the receiver can achieve maximum payoff no matter what the sender does, by mapping all signals to that cover-all act. Even if no one act is best in all states, there may be a cover-all act that works well for an agent nearly all the time. This is a within-agent matter. So we define 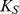 and 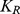, also making use of the Kendall tau distance. For each agent, we compare the preference orderings over acts that apply in different states of the world, comparing each pair of states in turn. K is high for an agent with respect to a pair of states if good acts in one state are bad acts in the other state. K for an agent averages all comparisons of states, rescaled to lie between zero and one, where 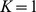 corresponds to the highest degree of contingency of payoff. (For details see Text S1.)

Our aim is to generalize about games with different levels of common interest and contingency of payoff for the agents. The method used is to generate samples from the space of games with three states where sender and receiver payoffs are integers between 0 and 99. Payoffs for each player for each act in a state are chosen randomly, so 18 random choices specify payoffs for a game. We then use the implementation of Lemke's [18] algorithm provided by the software package *Gambit* [19] to search for equilibria in that game where informative signals are being sent and used. The equilibrium concept used is the Nash equilibrium: a pair of strategies form a Nash equilibrium if neither player can improve their payoff by unilaterally modifying their strategy.

We measure the degree to which agents engage in informative signaling with *mutual information*, a symmetrical measure of the degree of association between two variables, measured in bits [20, p. 7]. An equilibrium is an *information-using* equilibrium if there is non-zero mutual information between states of the world and the receiver's acts. We focus on mutual information between states and acts for the following reasons. If there is mutual information between states and acts, the only way for this to arise is for senders to send informative signals and receivers to use these signals to guide variation in their actions to some extent. It is possible for senders to send signals with information about the state of the world that is not used – informative signals that are ignored by the receiver. It is possible also for receivers to guide actions with different signals sent randomly by the sender. The first of these – informative signals that are ignored – is a situation which may be an equilibrium and in which there is informative signaling, but it is not a situation in which the receiver is making use of that information. Our primary focus is situations in which informative signals are both sent and used. This requires that the signals carry information about states and acts carry information about signals. Given that receivers only have access to the state of the world by attending to signals, by the *data processing inequality* [20, p. 34] it is not possible for acts to carry more information about states than signals do. (States, signals, and acts form a Markov chain.) Any mutual information between states and acts arises from the use by the receiver of information about states in the signals.

Computational methods are described in Text S1 but one feature should be noted here: Lemke's algorithm is not guaranteed to find every equilibrium in a game [21]. So the reports of information-using equilibria below may be under-counts.

## Results

**Figure 1:**
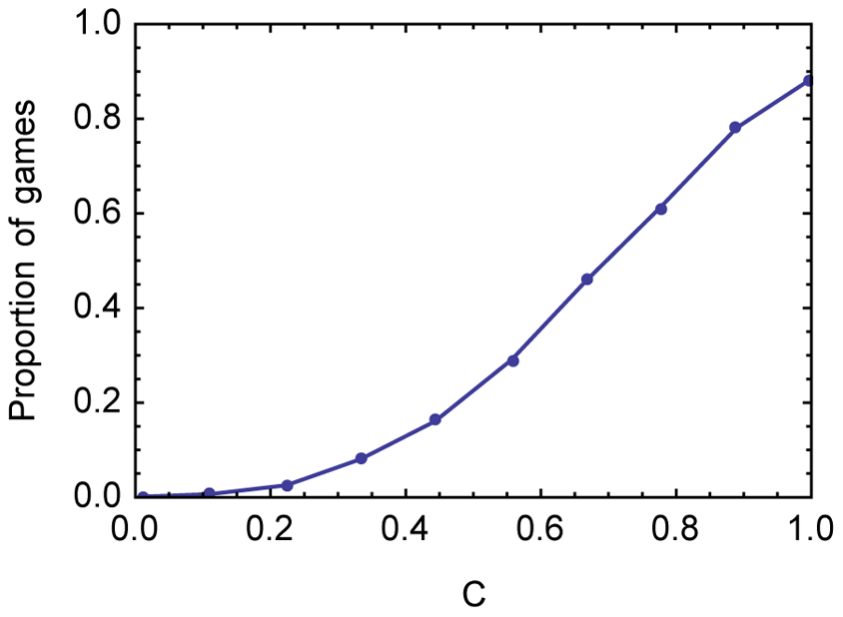
The proportion of games at each level of *C* with at least one information-using equilibrium. For each value of 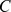, 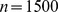.

To investigate the role of *C* we generated a random sample from the space of games with three equiprobable states, three receiver actions, and independently chosen payoffs for sender and receiver associated with each receiver action in each state of the world. (Each value of *C* is represented by 1500 games.) These sender and receiver payoffs are integers between 0 and 99. For each game we asked whether there is at least one information-using equilibrium in that game – an equilibrium with nonzero mutual information between states and acts – and then asked what proportion of games at each level of *C* have at least one information-using equilibrium. (All these games also have equilibria that are not information-using equilibria). The results are shown in Figure 1.

Very low degrees of *C* suffice to enable information-using equilibria, but at low *C* levels, only a small minority of games do so (unless the algorithm used has significant bias). As *C* increases, the fraction of games with information-using equilibria increases monotonically.

The curve in Figure 1 does not reach 100% for the case of complete common interest. Some games with 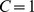 are games with zero 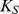 and 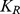. (When 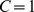, K is the same for sender and receiver.) The same act is best in every state. Around 1/9 games with 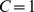 will also be 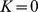. In such a game, the receiver can always take the system to an equilibrium by mapping all signals to the same, optimal, act. Then there is no mutual information between states and acts, regardless of what the sender is doing, as there is no variation in acts.

Surprisingly, a small number of games with 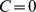, where sender and receiver have reversed preference orderings over acts in every state, have information-using equilibria. Table 2 shows a case of this kind – not a case from one of our samples, but a simplified case constructed using the computer-generated cases as a guide.

**Table 2:** A game with 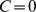 and an information-using equilibrium.

	*S*_1_	*S*_2_	*S*_3_
*A*_1_	5,5	2,4	2,1
*A*_2_	6,0	0,6	3,0
*A*_3_	0,6	6,0	0,3

Despite zero 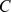, the game in Table 2 has an information-using equilibrium, whose sender and receiver rules are as follows:

**Sender:** 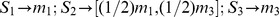


**Receiver:** 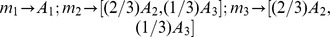


The mutual information between states and acts at this equilibrium is 0.67 bits, where the highest possible value for a game with three equiprobable states (a Lewisian signaling system) is 1.58 bits.

A feature of the case in Table 2 is that although sender and receiver have reversed preferences in every state, in 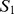 they share a second-best outcome (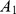) that is almost as good as their best. This is ignored by our measure 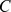, and it is one kind of common interest between the two agents. A way to modify *C* that takes this factor into account is to compare, across sender and receiver, their preference orderings over both the payoffs that arise from different actions and also the average of the payoffs for that agent in that state. This is done by defining a “dummy act” for the receiver in each state, an act that secures for each agent the mean of the other payoffs possible in that state. This dummy act and its payoff are then included in the determination of each agent's preference ordering over acts in that state; the two agents might agree, or disagree, for example, about whether the payoff of Act 1 is higher than the mean of their payoffs possible in that state. 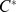, like 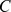, counts discordant pairs of preferences and is scaled to lie between 0 and 1. (For further details see Text S1). 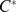 yields a similar relationship between common interest and the proportion of games with an information-using equilibrium to that seen in Figure 1.

The game in Table 2 has a nonzero 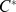, as sender and receiver agree about how one of their second-best outcomes compares to their means for that state, so 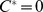 is a more demanding criterion for complete conflict of interest. Even in this stronger sense, though, it is possible for a game to have an information-using equilibrium with complete conflict of interest. A case of this kind, also one modeled on a less transparent computer-generated case, is shown in Table 3. This game has the following information-using equilibrium:

**Table 3:** A game with 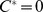 and an information-using equilibrium.

	*S*_1_	*S*_2_	*S*_3_
*A*_1_	1,8	8,1	0,6
*A*_2_	3,7	6,3	1,5
*A*_3_	8,1	1,8	5,3

**Sender:** 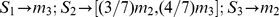


**Receiver:** 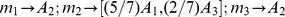


In all the cases with 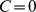 and/or 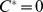 with information-using equilibria we have found, the underlying pattern is as follows. Two signals are used by the sender and three acts are used by the receiver. In one state the receiver produces an act that is intermediate in value for both sides. In the cases in Tables 2 and 3, this is 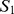. The receiver is prevented from shifting to their optimal act for this state by the fact that the signal sent in that state is ambiguous, and is sometimes also sent in a state for which the act that might “tempt” the receiver in 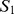 would be very bad. In another state, the receiver mixes their actions between optimal acts for each side. (This is 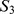 in both Tables 2 and 3.) Again, the receiver is prevented from settling on their optimal act in 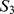 by the fact that the message the sender sends in that state is ambiguous; state 2 is used by the sender to deter exploitation in the other two states, and in this state all three acts are produced.

In both cases in Tables 2 and 3 the information-using equilbria are very fragile, as either the sender (in 3) or the receiver (in 2) can shift without penalty to a strategy in which the mutual information between states and acts goes to zero. Not all cases of information-using equilbria and zero common interest have this feature, however; sometimes information-use is less easily lost. The lowest level of common interest at which an information-using equilibrium is found in which neither sender nor receiver plays a mixed strategy, probabilistically varying their response to a state or a signal, is 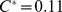 (see Text S1 for examples of both phenomena described in this paragraph).

A valuable feature of *C* is the weakness of the assumptions required for its measurement; *C* assumes only ordinal, not cardinal, utilities. 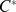 assumes cardinal utilities. 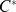 does not, however, assume that sender and receiver utilities are commensurable. If that further assumption is made, the notion of zero common interest can be analyzed instead by requiring that in every state, sender and receiver payoffs sum to a constant and the choice of action determines only how the division is made (a “constant-sum game”). We do not claim in this paper that information-using equilibria exist in constant-sum games. All constant-sum games have 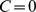, though the converse does not hold. Some constant-sum games have nonzero 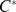, on the other hand, and not all 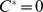 games are constant-sum. Due to its simplicity and weak assumptions, in the remainder of the body of this paper we will use *C* to measure common interest. 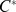 and constant-sum games are discussed in Text S1.

**Figure 2:**
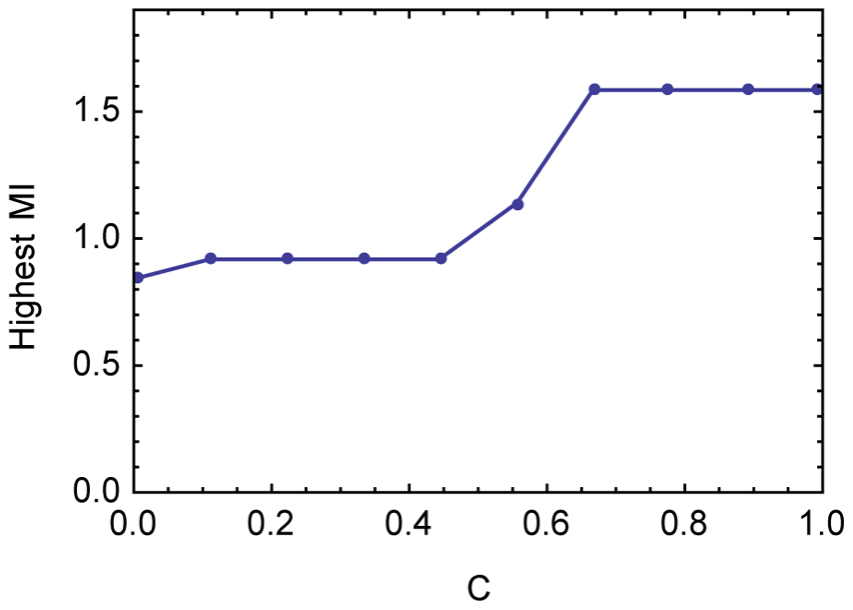
The highest level of information use at each level of 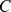. Measured in bits. For each value of 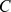, 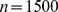.

Once we know how likely a given level of *C* is to maintain at least one information-using equilibrium, we can also ask what is the highest level of mutual information between states and acts that can be maintained in a game with a given degree of 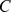. Figure 2 shows the maximum amount of mutual information between states and acts generated by an equilibrium pair of strategies from any game examined with a given level of 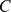. In constructing the pool of cases for this analysis, we have included not just the sample of games used in Figure 1 but also games found in earlier samples.

Figure 2 shows that the highest value for information use grows monotonically with common interest, as expected, but in a step-like way and with quite high values of mutual information between states and acts seen even at the lowest values of 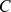. Conversely, our sample includes cases with high values of *C* and very minimal information use at equilibrium (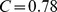, mutual information = 0.03 bits; see Text S1).

**Figure 3:**
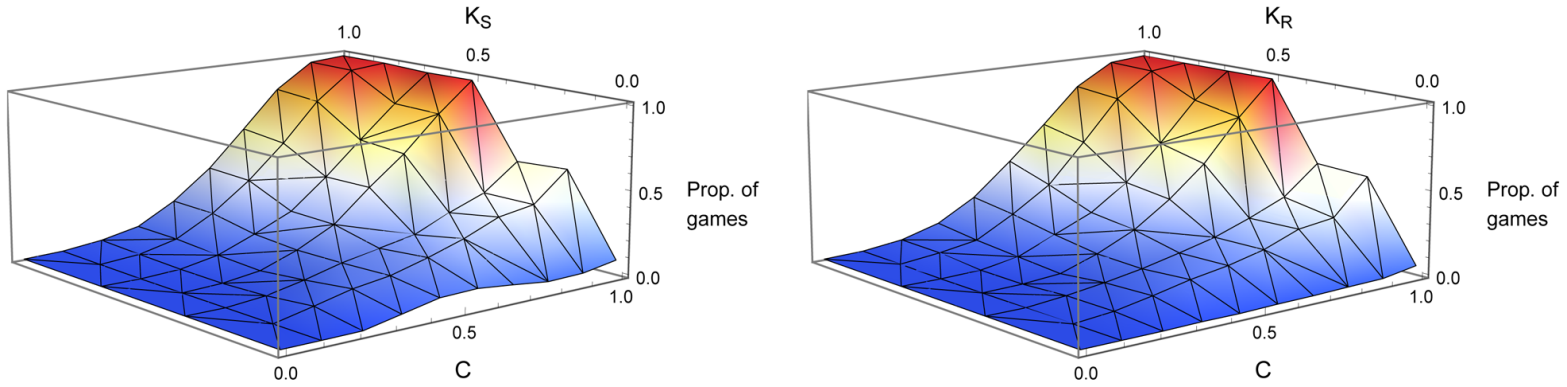
Relation between common interest, contingency of payoff for each agent, and the proportion of games with an information-using equilibrium. See Text S1 for explanations of C, 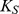 and 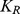. 1500 games were sampled and analyzed for each jointly possible combination of *C* and 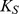 (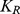).

A further analysis of these cases takes into account the contingency of payoff for sender and receiver, as well as common interest. The importance of this factor has been evident already in some extreme cases. When there is complete common interest but K is zero for both sides, there is no problem for signaling to solve – a single act always delivers an optimal payoff. When there is less common interest, the contingency of payoff for sender and receiver can diverge, and in most cases will be different. Figure 3 charts the proportion of games with at least one information-using equilibrium as a function of both common interest and contingency of payoff for an agent; separate graphs are given for 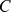 and 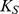 (left), and for 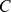 and 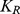 (right). The sample used for this chart is not the same one used for Figure 1, as a random sample of all games with a certain 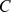 under-represents some combinations of 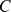 and 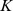. Figure 3 uses a sample in which every combination of 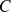 and 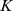 is represented by 1500 games.

As expected, higher values of 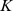 generate more information-using equilibria than lower values of 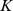. A difference is seen, however, between the consequences of low values of 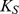 and 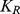. When the sender's contingency of payoff is very low, the intermediate values of 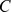 present a local maximum in the proportion of games with information-using equilibria. When 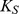 is low and 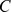 is intermediate, 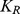 will be appreciable. The receiver seeks to vary their actions with the state of the world, and though the sender would ideally like the same act to always be performed, equilibria exist in which a compromise is reached. When the receiver's 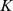 is low, on the other hand, they can achieve optimal payoffs by mapping every signal to the same act. The receiver can “go it alone” (though information-using equilibria arise in a few cases with high 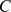 because of ties for the optimal act in a state).

## Discussion

We have given a treatment of the relation between informative signaling and common interest between sender and receiver, in a framework where signal use is associated with no differential costs and no role is given to iteration of interactions between agents. We find that informative signaling is possible in situations where sender and receiver have reversed preference orderings over receiver actions in every state of the world. This situation, where 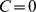, is one sense of “complete conflict of interest,” and a sense that has been employed more informally in a range of earlier discussions (eg., [22], [23]. In the light of our results, 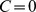 is shown to be a somewhat undemanding sense of complete conflict. We discussed one refinement of 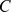, which requires stronger assumptions about payoffs, and found that information use at equilibrium is possible with complete conflict even in this stronger sense, where 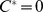. Another way to refine the idea of complete conflict, a way that uses still stronger assumptions, is by appeal to the notion of a constant-sum game. We do not claim that informative signaling is possible at equilibrium in constant-sum games. Another way to interpret our results is to suggest that the degree of conflict of interest in a game cannot be analyzed by noting the relationships holding between preferences in particular states, and then generalizing across states. Moving beyond consideration of these extreme values, we find that 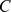 is a good predictor of the existence of information-using equilibria in the space of games studied in this paper.

We note several limitations of our model. First, the model assumes a particular relationship between sender and receiver, one where the sender has private knowledge of a state of the world, and payoffs result from the coordination of receiver actions with this state. This “state” of the world might be the condition or quality of the sender. Another kind of model assumes that neither side has privileged information about the state of the world, and the role of signaling is to coordinate acts with acts rather than acts with states (the “battle of the sexes,” for example). In further work we hope to extend our analysis to cover these cases. Another limitation involves our use of the Nash equilibrium concept. A Nash equilibrium need not be an evolutionarily stable strategy (because rivals may increase in frequency due to “drift”). In addition, equilibria of this kind may not be easily found by an evolutionary process [17]. Further work is needed to explore the dynamic properties of the games discussed in this paper. Thirdly, our analysis gives no role to the biological plausibility of games.

We close by comparing our treatment with two other papers, one classic and one recent. First, Crawford and Sobel [10] treated agreement in interests as a matter of degree, and found that when interests diverge, honest signaling is possible, but with lower informational content than there would be with complete agreement: “equilibrium signaling is more informative when agents' preferences are more similar.” In their model, the state of the world (sender quality) and the available actions both vary continuously in one dimension, and the difference between sender and receiver interests corresponds to a constant that is the difference between the actions seen as optimal by sender and by receiver in a given state of the world. In their model the degree of common interest across games can be measured exactly, but the model makes strong assumptions about the pattern of variation in the world. Our model makes weaker assumptions in this area, with the consequence that common interest is only partially ordered, motivating the introduction of coarse-grained measures such as *C* and 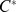. Crawford and Sobel found that as agents' interest converge, a larger number of distinct signals can be sent at equilibrium. We found that informative signaling can exist with zero common interest, through a combination of pooling and mixing, though games of this kind are rare and the proportion of games with an information-using equilibrium increases as interests converge. Crawford and Sobel's model also did not allow for variation in 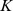, which we find has significant effects on the viability of information use.

Second, Zollman *et al.* [24] investigated biologically plausible games with two possible states of the world (again, sender quality) that are usually analyzed with substantial differential costs enforcing honesty. These authors found that very small differences in cost or benefit across different types of senders can maintain honest signaling when both sender and receiver mix strategies in a particular way. Senders in one state mix two signals, and senders in another state send just one of those signals. Receivers mix their responses to the ambiguous signal and do not mix their responses to the other. A conclusion from their model is that variation in signal-using behavior within a given situation, on both sender and receiver sides, need not be a matter of mere “noise” but can be an essential feature of an equilibrium state. Our results, within a framework of zero signal cost, lead to a conclusion of the same kind: probabilistic mixing of strategies, along with partial “pooling” of inputs, by both sign producers and sign interpreters can be important in maintaining signaling in situations of low common interest.

## Supporting Information

Text S1Methods – definitions – additional examples – 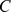, 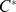, and constant-sum games – Interactions between common interest and contingency of payoff.(PDF)
