# Supplementary material for: Communication and Common Interest
Source: PLoS Comput Biol. 2013 Nov 7;9(11):e1003282. doi: 10.1371/journal.pcbi.1003282 (PMC3820505; doi:10.1371/journal.pcbi.1003282)
Supplement: Text S1 — Methods – definitions – additional examples – , , and constant-sum games – Interactions between common interest and contingency of payoff. (PDF) [file pcbi.1003282.s001.pdf]

Communication and Common Interest
Supporting Information

Peter Godfrey-Smith, Manolo Martínez
Philosophy Program, The Graduate Center, City University of New York
mmartinez3@gc.cuny.edu

September 25, 2013

Methods

The games analyzed in the text were generated randomly from the space of games with 3 equiprobable states, 3 messages, and 3 receiver actions. Payoffs were constrained to lie between 0 and 99. The sampling was carried out using the `randrange` function in the `random` module that is part of the Python standard library (version 3.3.2). The source code for this and all other calculations in this paper is available at https://github.com/manolomartinez/common-interest.

Formulas for the calculation of C, C^*, K_S, K_R, K_S^*, and K_R^* are given below. Note that tied payoffs are handled in the following way. When one agent has tied payoffs for two acts in a state, this is treated as a concordant pair in the calculation of the C parameters regardless of how the other agent orders those acts in that state. The treatment of ties in the calculation of the K parameters is analogous.

Measures of Common Interest and Contingency of Payoff

C: A measure of common interest between sender and receiver in a signaling game in which payoffs for both players depend on the pairing of the receiver's action with the state of the world. C measures the extent to which sender and receiver agree on their preference orderings over actions in each state.

For states $\{S_1, \dots, S_n\}$ and acts $\{A_1, \dots, A_n\}$, where the sender's payoff for act A_j in state S_i is α_{ij}, the receiver's payoff for act A_j in state S_i is β_{ij}, we define the function D_{sender}, that takes every triple of a state and two different acts to a rational:

$$D_{sender}\left(S_i, A_j, A_k\right) = \begin{cases} 0 & \text{if } \alpha_{ij} > \alpha_{ik} \\ 1 & \text{if } \alpha_{ij} < \alpha_{ik} \\ \frac{1}{2} & \text{if } \alpha_{ij} = \alpha_{ik} \end{cases}$$

$D_{receiver}$ is defined analogously:

$$D_{receiver}\left(S_i, A_j, A_k\right) = \begin{cases} 0 & \text{if } \beta_{ij} > \beta_{ik} \\ 1 & \text{if } \beta_{ij} < \beta_{ik} \\ \frac{1}{2} & \text{if } \beta_{ij} = \beta_{ik} \end{cases}$$

Finally,

$$C = 1 - \frac{2}{n(n-1)} \cdot \sum_{\substack{1\leq i\leq n,\\ 1<j\leq n,\\ 1\leq k<j}} P\left(S_i\right) \cdot \lfloor D_{sender}(i,j,k) - D_{receiver}(i,j,k) \rfloor$$

Some remarks regarding notation, which apply to all summations in this document:

- $\lfloor a \rfloor$ is the largest integer less than or equal to the absolute value of a.
- $\sum\limits_{\substack{a,\\ \dots\\ z}}$ is to be read as $\sum\limits_{a} \dots \sum\limits_{z}$.
- In this summation, k is always less than j. The intended effect is that when, e.g., $n = 3$, then $\langle j,k \rangle$ takes the values $\langle 2,1 \rangle$, $\langle 3,1 \rangle$, $\langle 3,2 \rangle$.

***C**:** A finer-grained measure of common interest. C^* measures the extent to which sender and receiver agree on their preference orderings over actions in each state, but tracks also whether sender and receiver agree that a given act in a state yields a payoff higher than the (unweighted) mean of the payoffs that the agent can receive in that state.

For each state S_i we introduce an extra payoff value for both sender and receiver:

$$\alpha_{in'} = \frac{1}{n} \sum_{1\leq j\leq n} \alpha_{ij}$$

$$\beta_{in'} = \frac{1}{n} \sum_{1\leq j\leq n} \beta_{ij}$$

where $n' = n + 1$. Then,

$$C^* = 1 - \frac{2}{n'(n'-1)} \cdot \sum_{\substack{1\leq i\leq n,\\ 1<j\leq n',\\ 1\leq k<j}} P\left(S_i\right) \cdot \lfloor D_{sender}(i,j,k) - D_{receiver}(i,j,k) \rfloor$$

K_S and K_R: K_S (K_R) measures the extent to which the sender's (receiver's) preference ordering over receiver actions varies with the state of the world.

With D_{sender} and $D_{receiver}$ as above,

$$K_S = \sum_{\substack{1<i\leq n,\\ 1\leq j<i,\\ 1<k\leq n,\\ 1\leq l<k}} \frac{2}{n(n-1)} \cdot \lfloor D_{sender}(i,k,l) - D_{sender}(j,k,l) \rfloor$$

$$K_R = \sum_{\substack{1<i\leq n,\\ 1\leq j<i,\\ 1<k\leq n,\\ 1\leq l<k}} \frac{2}{n(n-1)} \cdot \lfloor D_{receiver}(i,k,l) - D_{receiver}(j,k,l) \rfloor$$

These formulas are not normalized. In the generation of data for this paper, the normalization coefficients (i.e., the maximum values for K_S and K_R) were found numerically.

K_S^* and K_R^*: These are modifications of measures K_S and K_R. The modification is strictly analogous to the one that takes C to C^*.

With $D_{sender}, D_{receiver}, n', \alpha_{in'}, \beta_{in'}$ as above,

$$K_S^* = \sum_{\substack{1<i\leq n,\\ 1\leq j<i,\\ 1<k\leq n',\\ 1\leq l<k}} \frac{2}{n(n-1)} \cdot \lfloor D_{sender}(i,k,l) - D_{sender}(j,k,l) \rfloor$$

$$K_R^* = \sum_{\substack{1<i\leq n,\\ 1\leq j<i,\\ 1<k\leq n',\\ 1\leq l<k}} \frac{2}{n(n-1)} \cdot \lfloor D_{receiver}(i,k,l) - D_{receiver}(j,k,l) \rfloor$$

Again here, these formulas are not normalized. As for K_S and K_R, the normalization coefficients were found numerically.

Additional Examples

The games represented in Tables 2 and 3 of the main text are modeled on games appearing in our computer-generated samples. Above are two games from that sample, with $C = 0$ (Table S1a) and $C^* = 0$ (Table S1b).

In both these games – and in contrast to the games in Tables 2 and 3 – it is *not* the case that either sender or receiver, merely by deploying pure strategies, can eliminate information use. Once in an information-using equilibrium, no possible sequence of pure-strategy best responses by sender or receiver can take either of them to a strategy in which there is complete pooling (where A is a best response to B if and only if there is no response to B that gains a higher payoff than A). So no amount of "drift" in these cases can take sender and receiver from a situation in which information is used to a situation in which it is not.

The game with lowest common interest uncovered in our sample in which there is an information-using equilibrium where neither sender or receiver uses a mixed strategy is a game in which $C = 0.22$ and $C^* = 0.11$. This game is in Table S2a. Table S2b gives another example discussed in the text, a case where common interest is high ($C = 0.78$) and mutual information between states and acts is present at an equilibrium, but the amount of mutual information is very small: 0.03 bits.

	S_1	S_2	S_3
A_1	44,9	21,69	84,49
A_2	14,71	85,4	38,56
A_3	25,16	35,44	83,55

Sender: $S_1 \to m_2; S_2 \to [(3/65)m_1, (62/65)m_2]; S_3 \to m_1$

Receiver: $m_1 \to A_3; m_2 \to [(25/32)A_1, (7/32)A_2]; m_3 \to [(25/32)A_1, (7/32)A_2]$

(a) A game with $C = 0$

	S_1	S_2	S_3
A_1	31,7	0,95	57,26
A_2	5,71	99,1	15,62
A_3	17,66	62,23	28,48

Sender: $S_1 \to m_1; S_2 \to [(29/47)m_1, (18/47)m_2]; S_3 \to m_2$

Receiver: $m_1 \to A_3; m_2 \to [(37/99)A_1, (62/99)A_2]; m_3 \to [(37/99)A_1, (62/99)A_2]$

(b) A game with $C^* = 0$

Table S1: Games and their information-using equilibria

	S_1	S_2	S_3
A_1	17,27	22,72	87,1
A_2	38,15	75,16	70,38
A_3	18,29	8,45	81,12

Sender: $S_1 \to m_2; S_2 \to m_3; S_3 \to m_3$

Receiver: $m_1 \to A_3; m_2 \to A_3; m_3 \to A_1$

(a) A game with $C = 0.22$ ($C* = 0.11$) and a pure-strategy information-using equilibrium

	S_1	S_2	S_3
A_1	92,79	23,59	99,64
A_2	68,57	40,80	22,86
A_3	7,41	23,66	28,64

Sender: $S_1 \to m_2; S_2 \to m_3; S_3 \to m_2$

Receiver: $m_1 \to A_3; m_2 \to [(6/77)A_1, (71/77)A_2]; m_3 \to A_2$

(b) A game with $C = 0.78$ and mutual information between states and acts of 0.03 bits.

Table S2: Games and their information-using equilibria

C, C^*, and constant-sum games

Our approach to measuring common interest attends to the relations between the sender's and receiver's preference orderings over actions in each state (see above for details). C assumes only ordinal utilities, while C^* assumes cardinal utilities but does not require that sender and receiver payoffs be commensurable. An alternative concept of complete conflict of interest uses the idea of a constant-sum game: a game is constant-sum if for each state there is a total payoff available that is divided in different ways between the two agents, depending on the receiver's action. Actions affect the division but not the amount divided. This concept of conflict of interest assumes cardinal utilities and commensurability across agents. This commensurability condition is not met in some biologically relevant cases, such as those in which signaling occurs across partners from different species – often, different kingdoms – and these are cases where the concept of common interest can be fruitfully applied [1, 2, 3]

When the stronger set of assumptions required to classify games as constant-sum, or not, are met, the following implications hold. All constant-sum games have $C = 0$, but not conversely. All $C^* = 0$ games have $C = 0$, but not conversely. Some constant-sum games are $C^* = 0$ and some are not, as a consequence of the role played by ties (see above; when sender and receiver in a constant-sum three-act game agree, for example, that their middle score is midway between their high and low scores in a state, this gives them non-zero C^*, as was pointed out to us by Elliott Wagner). Lastly, not all $C^* = 0$ games are constant-sum.

As noted in the text, the function relating the proportion of games with an information-using equilibrium to C^* is similar to the function for C. Figure S1 below, analogous to Figure 1 in the main text, gives the proportion of games at each level of C^* with at least one information-using equilibrium.

Interactions between common interest and contingency of payoff

Figure 3 in the main text shows interactions between common interest (C) and contingency of payoff for sender (K_S) and receiver (K_R) . As expected, the proportion of games with information-using equilibria generally increases with C and with both K_S and K_R. Contingency of payoff for sender has a more complex role, however, as when the sender's contingency of payoff is very low, intermediate values of C present a local maximum in the proportion of games with

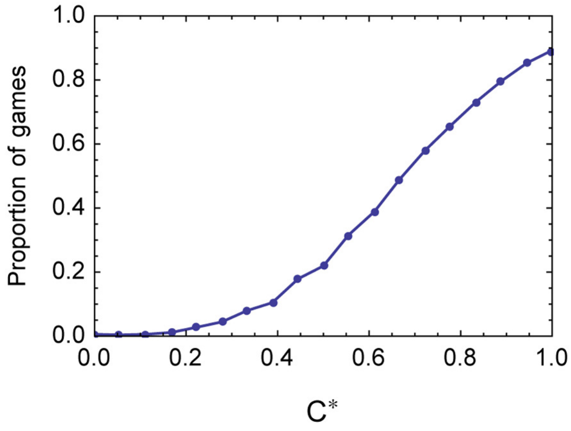

Figure S1: The proportion of games at each level of C^* with at least one information-using equilibrium. For each value of C^*, $n = 1500$.

information-using equilibria. The asymmetry between the roles of sender and receiver has the consequence that a sender can benefit from sending informative signals even when $K_S = 0$, as they need to influence a receiver who seeks to vary their act with the state of the world.

We performed a finer-grained analysis of these relationships by using C^* rather than C, and K_S^* and K_R^*. These latter measures are similar to K_S and K_R but are adjusted in the same way that C^* adjusts C. K_S^*, for example, compares the preference orderings over acts for the sender in different states of the world, but also compares the payoff for each act to the mean of the different payoffs the sender might receive in that state. (Computational formulas are above.) The aim here, as in C^*, is to track cases where two states share a second-best act whose payoff is close to the best possible payoff in those states. The results are given in Figure S2 below. The resulting analysis gives similar results to those discussed in the text, with a somewhat clearer "hump" in the sender's chart at low values of K_S^* and intermediate values of C^*.

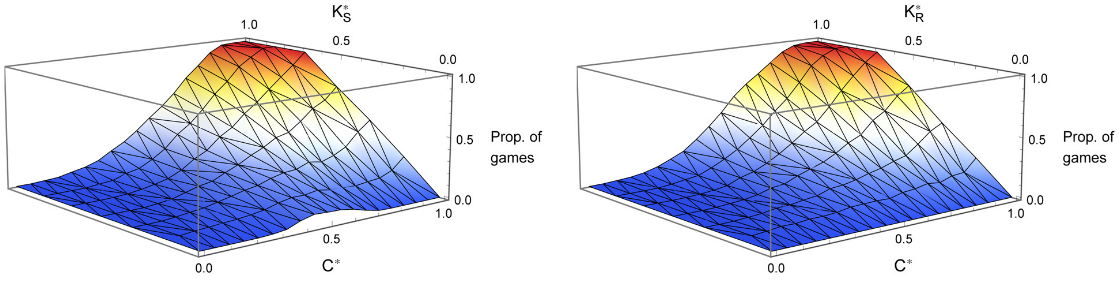

Figure S2: Relation between common interest (using C^*) contingency of payoff for each agent (using K_S^* and K_R^*), and the proportion of games with an information-using equilibrium. 1500 games were sampled and analyzed for each jointly possible combination of C^* and K_S^* (K_R^*).

References

[1] Bergstrom, C. and Lachmann, M. (2001). Alarm calls as costly signals of antipredator vigilance: The watchful babbler game. *Animal Behavior*, 61:535–543.

[2] FitzGibbon, C. D. and Fanshawe, J. H. (1988). Stotting in thomson's gazelles: an honest signal of condition. *Behavioral Ecology and Sociobiology*, 23:69–74.

[3] Harrison, M. J. (2005). Signaling in the arbuscular mycorrhizal symbiosis. *Annual Review of Microbiology*, 59:19–42.
